# Chromosomal homology of *Uraeotyphlus oxyurus* group of species (Amphibia, Gymnophiona, Ichthyophiidae)

**DOI:** 10.3897/CompCytogen.v7i1.3603

**Published:** 2013-03-18

**Authors:** G. Venu, G. Venkatachalaiah

**Affiliations:** 1 Centre for Applied Genetics, Department of Zoology, Bangalore University, Bangalore 560 056, India

**Keywords:** *Uraeotyphlus oxyurus* species group, karyotypes, chromosomal homology

## Abstract

*Uraeotyphlus oxyurus* (Dumeril et Bibron, 1841), *Uraeotyphlus interruptus* Pillai et Ravichandran, 1999, *Uraeotyphlus narayani* Seshachar, 1939 and *Uraeotyphlus menoni* Annandale, 1913 were cytogenetically analysed following conventional and differential staining techniques. These species show similar karyotypes with 2n=36 (FN=58). There were no traces of species-specific features in regard to C-banding and NOR staining. The comparative study of karyotypes shows chromosomal homologies among the four species. Chromosomal data seem to support the concept that two species groups exist in the genus *Uraeotyphlus*.

## Introduction

The genus *Uraeotyphlus* Peters, 1879is endemic to the Western Ghats region of peninsular India and constitutes one of the three genera within the family Ichthyophiidae Taylor, 1968 along with *Caudacaecilia* Taylor, 1968 and *Ichthyophis* Fitzinger, 1826(Wilkinson et al. 2011). Its taxonomy had been uncertain till the publication of Nussbaum and Wilkinson (1989) which gave this group a family level status among the existing caecilians of India. However, after a lapse of a decade or so, this prevailing situation seemed to have recovered moderately and is sufficient in redefining interrelationships among other families of caecilians based on morphological and molecular evidence (Wilkinson and Nussbaum 1996, 2006, Frost et al. 2006, Gower and Wilkinson 2007, Roelants et al. 2007, Gower et al. 2008, Zhang and Wake 2009, Wilkinson et al. 2011).

On the basis of morphological features such as cylindrical body, annulation and of the presence or absence of phallodeum, and of limited molecular evidence, Gower and Wilkinson (2007) partitioned this supposedly monophyletic genus *Uraeotyphlus* into two species groups: *Uraeotyphlus oxyurus* species complex and *Uraeotyphlus malabaricus* species group. Consequent upon this arrangement, *Uraeotyphlus oxyurus* (Dumeril & Bibron, 1841), *Uraeotyphlus interreptus* Pillai & Ravichandran, 1999, *Uraeotyphlus narayani* Seshachar, 1939 and *Uraeotyphlus menoni* Annandale, 1913 were grouped as derived species, while of *Uraeotyphlus gansi* Gower, Rajendran, Nussbaum & Wilkinson, 2008, *Uraeotyphlus oommeni* Gower & Wilkinson, 2007 and *Uraeotyphlus malabaricus* (Beddome, 1870) were considered as primitive ones (Gower et al. 2008).

Earlier, Seshachar (1939) presented the male meiotic chromosomal complement of *Uraeotyphlus narayani* with the dipoloid number of 36 and gave detailed descriptions based on chromosomal morphs observed such as V-shaped, rods and dots in the complement. Elayidom et al. (1963) have described the somatic and meiotic chromosomal complement of *Uraeotyphlus menoni* as consisting of diploid number of 36 for the species, but neither study presented karyotypic characteristics. Venkatachalaiah and Venu (2002) gave a detailed karyotypic characteristic of what was then mistakenly thought to be *Ichthyophis malabarensis* Taylor, 1960 (2n=36, FN= 60) but which was subsequently found to be *Uraeotyphlus* prope *interruptus* of Ichthyophiidae. With this surge of interest to elucidate the phylogeny and evolution, Venu et al. (2011) have presented the chromosomes of a member of the *Uraeotyphlus malabaricus* species group, *Uraeotyphlus gansi*, bearing2n=42, FN=58, highest diploid number known thus far for a member of the genus *Uraeotyphlus* of the family Ichthyophiidae.

In this study, we present the karyotypes of *Uraeotyphlus oxyuryus* and *Uraeotyphlus interruptus* and the results of reanalysis of chromosomes of *Uraeotyphlus narayani* and *Uraeotyphlus menoni* with a view to providing new insights into the intragenus relationships within the genus *Uraeotyphlus*.

## Material and methods

Specimens of both sexes, collected from different regions the Western Ghats (Table 1) a few days before the experiment, were kept in glass aquaria under suitable conditions. After *in vivo* colchicine treatment, chromosome preparations were obtained from the liver, the gut epithelium and the testis. Cell suspensions, hypotonic treatment and fixation of cells were performed as described earlier (Venkatachalaiah and Venu 2002, Venu et al. 2011). Chromosome number and standard karyotype (in respect of somatic metaphase and meiotic pachytene) morphology were determined by conventional Giemsa staining technique. Chromosome nomenclature was followed as proposed earlier by Levan et al. (1964) but adopted for the present situation as described earlier (Venkatachalaiah and Venu 2002).

Conventional C-banding was performed according to Sumner (1972) using Ba(OH)_2_ at 60^0^C followed by staining in dilute Giemsa solution, with modifications in alkaline treatment.

Location of nucleolus organizer regions (NORs) was performed by applying the one-step silver nitrate method of Goodpasture and Bloom (1975).

**Table 1.** Details of collection of *Uraeotyphlus interruptus*, *Uraeotyphlus narayani*, *Uraeotyphlus menoni* and *Uraeotyphlus oxyurus*

**Species**	**Locality**	**Habitat**	**Voucher number**	**No. of animals used**	**Geographical coordinates**
*Uraeotyphlus interruptus*	Gudalur, Nilgiris (Dt), Tamil Nadu, India	Mixed plantations of tea, banana, pepper, orange, coffee	BUB114, 103BUB105, 111	2 males2 females	11°30'0"N, 76°30'00"E
*Uraeotyphlus narayani*	Changanssery,Kottayam (Dt), Kerala, India	Backyard garden with banana plantation	BUB101, 115BUB109, 116	2 males2 females	9°28'00"N, 76°33'00"E﻿
*Uraeotyphlus menoni*	Mattathur, Thrissur (Dt), Kerala, India	Backyard garden with banana plantation	BUB107, 113BUB106, 102	2 males2 females	10°22'45"N, 76°19'15"E
*Uraeotyphlus oxyurus*	Agali, Palakkad (Dt), Kerala, India	Cultivated agricultural land with banana and coconut plantation	BUB104, 112BUB108, 110	2 males2 females	11°5'0"N, 76°35'0"E

## Results

### Karyotypes of *Uraeotyphlus oxyurus* and *Uraeotyphlus interruptus*

**Figure 1.**
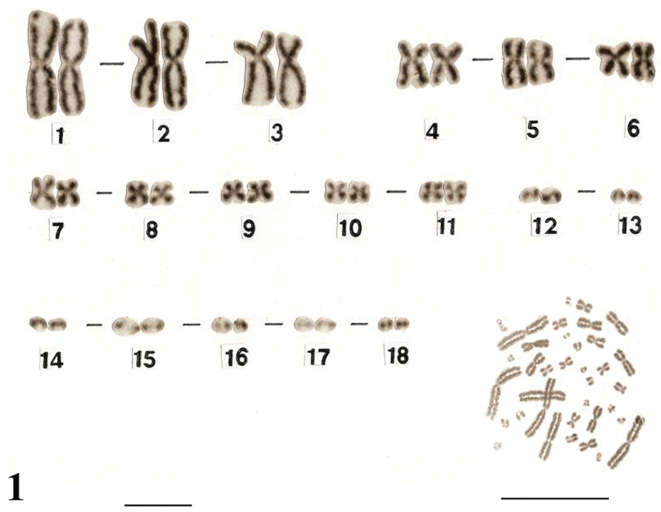
Giemsa stained male karyotype and female metaphase complement of *Uraeotyphlus oxyurus*. Bar = 10 µm.

**Figure 2.**
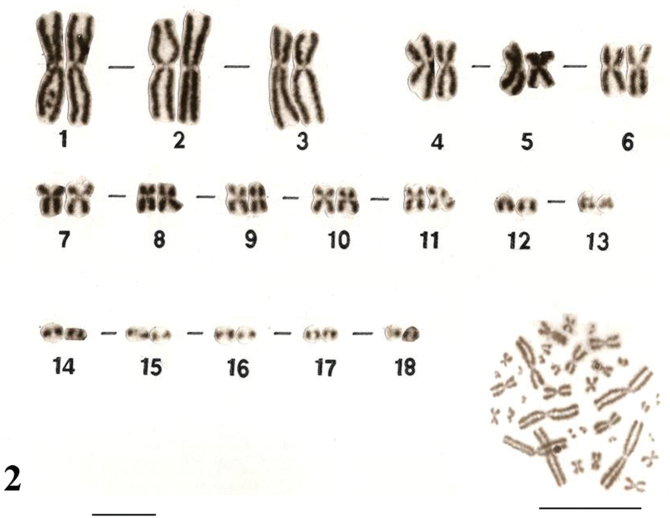
Giemsa stained male karyotype and female metaphase complement of *Uraeotyphlus interruptus*. Bar = 10 µm.

The karyotypes of *Uraeotyphlus oxyurus* (Fig. 1) and *Uraeotyphlus interruptus* (Fig. 2) revealed a diploid chromosomal complement consisting of 2n=36, FN=58.

The somatic metaphase chromosomes in the karyotype could be divided into four groups, A, B, C and D, based on the decreasing order of total length and position of centromere of each chromosome. The first group (A) includes two pairs (1–2) of large metacentrics and one pair (3) of submetacentrics, while the B group consisted of three pairs (4–6) of medium sized metacentrics in which the pair four was slightly longer than other two pairs. The third group (C) included 5 pairs (7–11) of smaller submetacentrics, all in decreasing order of their total length. The fourth group (D) included mostly acrocentric (12–18) pairs.

**Figure 3.**
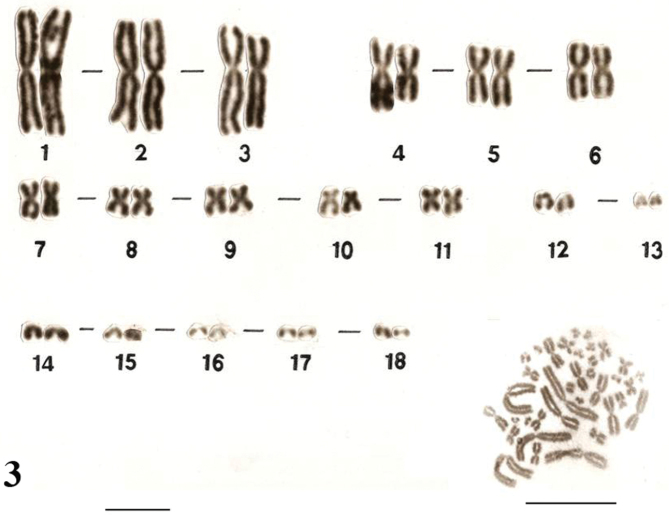
Giemsa stained male karyotype and female metaphase complement of *Uraeotyphlus narayani*. Bar = 10 µm.

**Figure 4.**
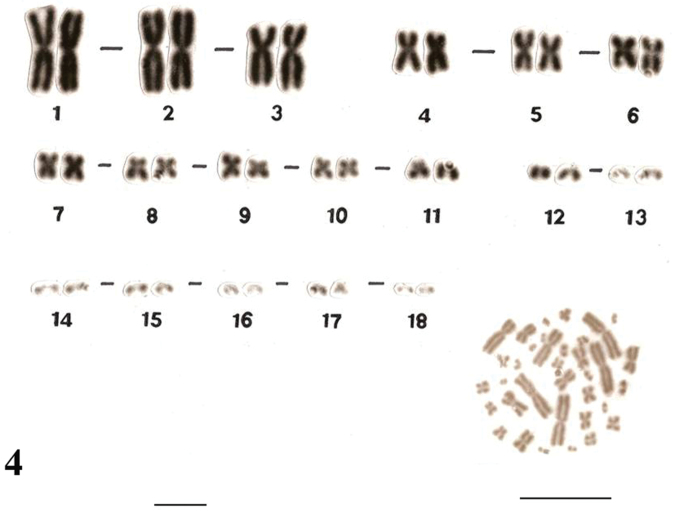
Giemsa stained male karyotype and female metaphase complement of *Uraeotyphlus menoni*. Bar = 10 µm.

Similar karyotypes were obtained for the species *Uraeotyphlus narayani* (Fig. 3) and *Uraeotyphlus menoni* (Fig. 4).

No detectable sex chromosome pair was observed in either sex in the metaphase chromosomal complement and karyotypes of *Uraeotyphlus oxyurus*, *Uraeotyphlus interruptus*, *Uraeotyphlus narayani* and *Uraeotyphlus menoni*.

### Meiosis

**Figure 5.**
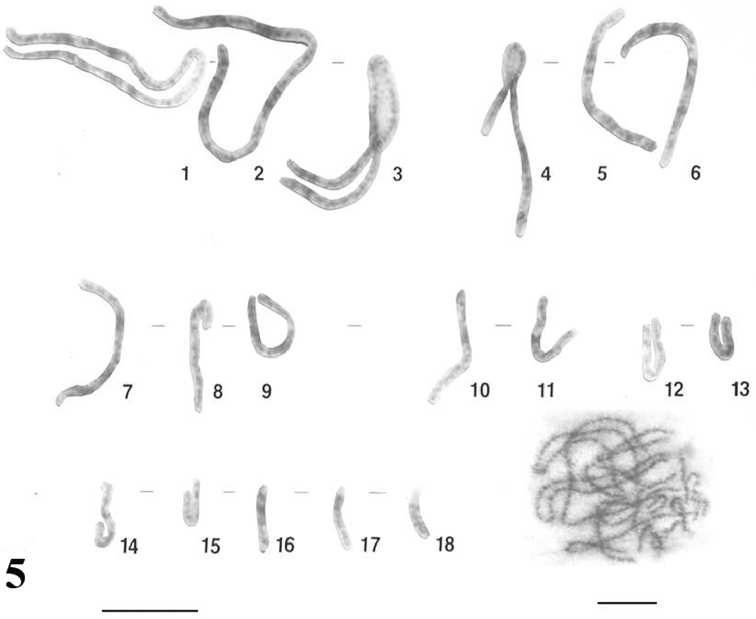
Pachytene karyotype and complement of *Uraeotyphlus oxyurus*. Bar = 10 µm.

**Figure 6.**
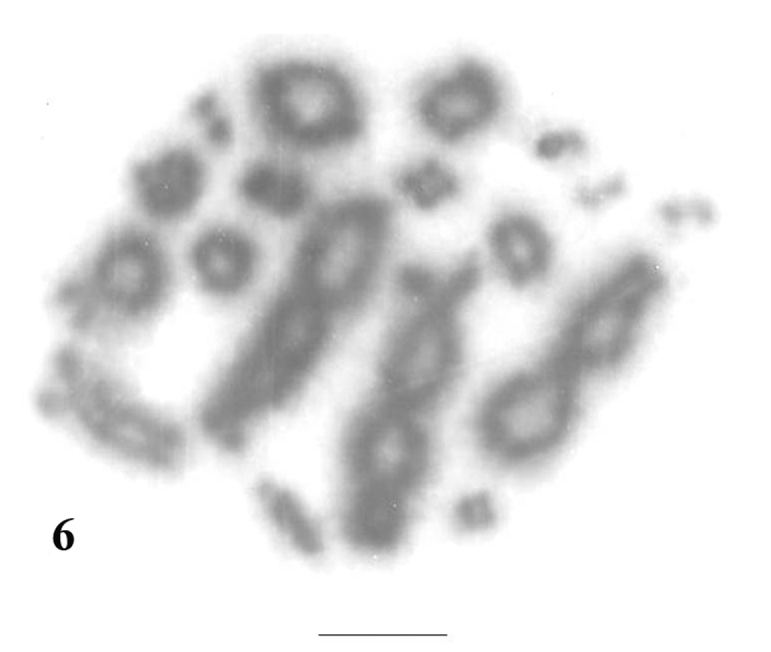
Diplotene complement of *Uraeotyphlus oxyurus*. Bar = 10 µm.

The meiotic chromosomes prepared from male individuals of *Uraeotyphlus oxyurus* revealed a good number of pachytene (Fig. 5), diplotene (Fig. 6), diakinetic and second meiotic metaphase configurations.

Pachytene chromosome karyotype constructed as per the somatic metaphase chromosome karyotype revealed eighteen pachytene bivalents corresponding the eighteen pairs of somatic chromosomes.

The diplotene complement allowed the counting of eighteen individually identifiable bivalents. In each diplotene complement, the longer ones carried 4-6 chiasmata, whereas the smaller acrocentrics consisting of at least one chiasma.

Similar results were obtained for the other three species, *Uraeotyphlus interruptus*, *Uraeotyphlus narayani* and *Uraeotyphlus menoni*.

## C – Staining

**Figure 7.**
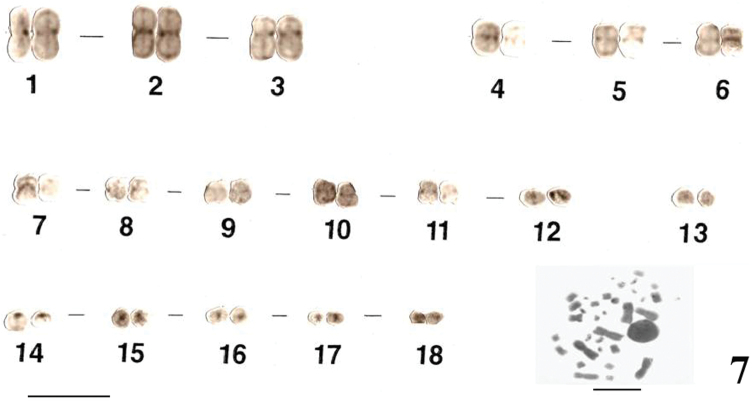
C-stained male karyotype and female metaphase complement of *Uraeotyphlus oxyurus*. Bar = 10 µm.

The *Uraeotyphlus oxyurus* karyotype is characterized by discernible but faintly stained centromeric C-bands in the metacentric and submetacentric chromosomes, while the acrocentrics have very prominent C-bands at the centromeric region cumulatively highlighting both the centromeric regions and the proximal portions of each short arms of each chromosome (Fig. 7). In comparison with the C-staining characteristics of *Uraeotyphlus oxyurus*, typical C-bands were observed in the other three species karyotypes.

### Ag-NOR – Staining

**Figure 8.**
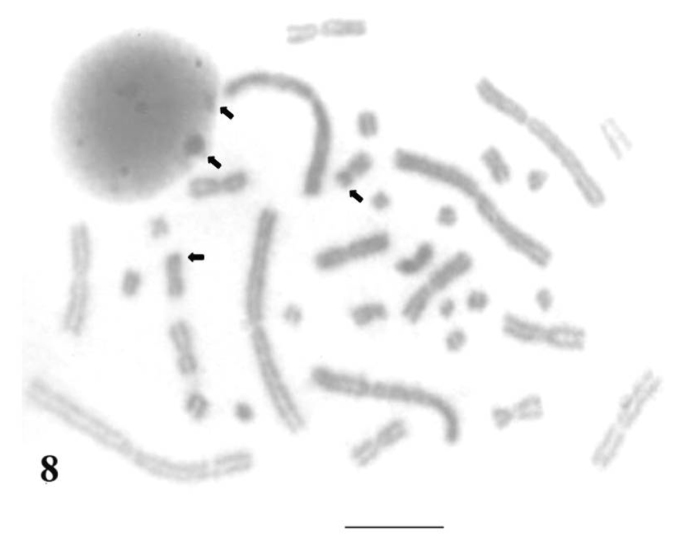
Silver-stained interphase nucleus and mitotic metaphase complement of *Uraeotyphlus oxyurus*. Bar = 10 µm.

Silver nitrate staining showed that in *Uraeotyphlus oxyurus*, Nucleolus Organizer Regions (NORs) are confined to chrormosomal pair 9 in the complement but were not consistently demonstrated in each and every cell. Whereas, interphase nuclei demonstrated 1 to 2 (or sometimes 3) numbers of silver nitrate (2–3) aggregates (Fig. 8). This situation is perhaps an indication of limited proportionality of rDNA sites that could not be elicited cytologically and those of transcriptionally silent NORs did not form any discrete Ag-NORs sites at metaphase chromosomes.

Similar kind of results could be drawn for the other three species, *Uraeotyphlus interruptus*, *Uraeotyphlus narayani* and *Uraeotyphlus menoni*.

## Discussion

Emphasizing on systematics of those Indian endemic uraeotyphlids, the morphological attributes as elicited by Gower and Wilkinson (2007) and Gower et al. (2008) are in congruence with the results of cytogenetic data that were available for determining their primacy and prevalence of bimodal karyotypic characteristics.

In the present study, the karyotype carrying a diploid number of 36 (2n=36) chromosomes was found consistently identical in each of the four species belonging to *Uraeotyphlus oxyurus* species complex. The cytogenetic data converged on chromosome morphology indicate that the genus *Uraeotyphlus* is relatively well conserved with most chromosomal pairs classified as meta- and submetacentrics in size and shape. This observation, especially of all the four *oxyurus* type karyotypes having a homologous situation reveals a closer phylogenetic relationship. This, in turn, supports consideration for a monophyletic origin.

The same situation cannot be considered as applying to those species belonging to *Uraeotyphlus malabaricus* group. Until now, none of these species karyotypes were known except a publication citing a variable karyotype for the species belonging to the genus, *Uraeotyphlus*, *Uraeotyphlus gansi*. Venu et al. (2011) have described the karyotype of *Uraeotyphlus gansi* bearing a different basic chromosomal number (2n=42) and morphology. A major attribute of *Uraeotyphlus gansi* karyotype is that it is similar to any of the known karyotypes of the species belonging to *Ichthyophis* and *Caudacaecilia* of the family Ichthyophiidae (Venkatachalaiah and Venu 2002, Matsui et al. 2006, Venu 2008, Venu et al. 2011).

This karyolgical description lends support to Gower and Wilkinson’s (2007) contention that it is possible to draw conclusions as to the karyological affinities between the two taxa through a comparison of the primitive uraeotyphlid karyotype with that of ichthyophiid karyotypes. The karyogrammic and morphometric data on the karyotype of *Uraeotyphlus gansi* and that of representative karyotype of *Ichthyophis* and *Caudacaecilia* (Nussbaum 1991; Venkatachalaiah and Venu 2002; Matsui et al. 2006; Venu 2008) lend support to the concept of closer phylogenetic affinity between the two taxa within the family (Wilkinson et al. 2011, Venu et al. 2011).

The four species karyotypes (2n=36) may be considered as derived ones from that of *Uraeotyphlus gansi* karyotype (2n=42), which serves as a modal karyotype for the *Uraeotyphlus malabaricus* group species which could be considered a basal one among uraeotyphlids. Besides, based on the pronounced chromosomal homogeneity among the said species groups’ karyotypic specificities, this may be considered as of cytogenetic importance while placing emphasis on species differentiation within the genus. Uraeotyphlid chromosome complements belonging to family Ichthyophiidae seem to present bimodal diploid numbers: 2n=42 chromosomes in the basal type *Uraeotyphlus malabaricus* group and 2n=36 chromosomes in the case of derived species belonging to *Uraeotyphlus oxyurus* species complex.

Presently, chromosomal data on ichthyophiid taxa indicate that they are all well-conserved among the species whose karyotypes are known, bearing identical chromosomal set in each case (Venkatachalaiah and Venu 2002; Venu 2008). The characteristics of the four *Uraeotyphlus oxyurus* karyotypes and of *Uraeotyphlus gansi* karyotype are unique in possessing most chromosomal pairs as conserved. The centromeric position of the lowerset of chromosomes in the karyotype makes all the more significant for consideration of their karyotypic specificities.

During the course of drawing karyological relationships prevailing between the two taxa (for e.g., ichthyophiids and uraeotyphlids) it is possible to infer that most submeta and metacentrics are conserved to a maximum extent and only acrocentrics mayhave paved the way for chromosome speciation events.

There are instances that exhibit very little variation in their karyological features. Marked karyological homogeneity seems prevalent in many taxa of cyprinid fishes (Caputo et al. 2003, Rabova et al. 2003, Mesquita et al. 2008) and in some salamanders (Sessions 2008) and anuran amphibians (Kasahara et al. 2003, Aguiar et al. 2004, Rodrigues et al. 2011) and evidently in cryptodire reptilians (Olmo 2005, Castiglia et al. 2009). Karyological uniformity as demonstrated by some of these studies (including the present report) seems to point towards eliciting closer affiliation in their respective lineages. Moreover, this kind of situation in the context of phylogenetic assessment reflects upon their initial stages of evolutionary consequences.

Any attempt on comparison of karyotypes from the present study to that of other representative karyotypes for *Uraeotyphlus malabaricus* type (although only one species karyotype is available) makes clear the occurrence of a succession of chromosomal rearrangements, mainly through pericentric inversion and / or fusion. This appears necessary in order to create the karyotype found in described karyotype from those of ancestral ones. This sequence of events could account for the appearance of reduction in basic chromosome numbers from 2n=42 chromosome to 2n=36 chromosomes (Venkatachalaiah and Venu 2002).

The C-banding profile and NOR localization seems to be a homologous feature in *oxyurus* uraeotyphlids. C-banding pattern was found identical in the four species of *oxyurus* group within the genus. The variation illustrated by the two species group (viz., *oxyurus* and *malabaricus*) in which there is an enormous interspecific variation was evident in the distribution and amount of heterochromatin (Venu 2008; Venu et al. 2011). The NOR location is a conserved characteristic within the *oxyurus* group species. This view is in accordance with that of Schmid et al. (1990) opinion that in closely related species, the NORs are always almost located in the same chromosome regions within the complement.

Based on certain molecular analysis, it is possible to ascribe that the basal *Uraeotyphlus malabaricus* group is closely aligned in its affinity to primitive ichthyophid lineages. However, in order to expend linealogical connections between these two broader groups, Gower et al. (2002) and Frost et al. (2006) have proposed a possibility of an intermediate taxon.

Phylogeny of the Indian endemic genus *Uraeotyphlus* is still poorly known. But a combined approach based on morphology, biochemistry, molecular biology and cytogenetics might help to resolve a revised classification of Ichthyophiidae and thus to understand better of their phylogenetic relationships with other caecilians. Towards that effect, San Mauro et al. (2004) have attempted to provide intrafamily relationships of extant caecilians mainly based on mitochondrial genomes since they found them offering a more reliable data set for comparison. Subsequently, Zhang and Wake (2009) have extended this type of molecular analyses to include multiple gene data sets and more number of taxa. Their work on the key species *Ichthyophis malabarensis* uncovered findings favoring sisterly relationships between the genera i.e., *Ichthyophis* and *Uraeotyphlus* and thereby supporting the view that they are to a certain extent, paraphyletic in nature. The latter study have offered support to the proposition of Gower and Wilkinson (2007) that the genus *Uraeotyphlus* may be divided into the plesiomorphic *Uraeotyphlus malabaricus* group and apomorphic *Uraeotyphlus oxyurus* group. The results of present study strengthen support to the derived status of *Uraeotyphlus oxyurus* group species.

Maddin et al. (2012) have attempted to explore the possibility of utilizing morphology based upon microcomputed tomographic pictures of brain case and stape of several caecilian taxa, as an additional criterion to other morphological features that ascribed during the course of their phylogenetic assessment. However, this study seems to point towards reaching congruence thereby limiting its extent to generic level classification but not at species specificity.

While exploring probable phylogenetic relationships and rapport, it also seems possible to infer that karyological data fall in line with those of recently assimilated molecular analyses (San Mauro et al. 2004, Zhang and Wake 2009).

In conclusion, the cytogenetic study based on conventional Giemsa staining including C- and NOR bandings, upon four *oxyurus* group species of *Uraeotyphlus* taxa, indicate that they are more similar in their karyotypic profile (if not identical) which might form a monophyletic group. In the light of extensive chromosomal homology incurred, that does not preclude minor karyotypic differences necessitating in the use of other banding techniques for the improvement of karyological characterization.
